# Whole Genome Sequencing for the Retrospective Investigation of an Outbreak of Salmonella Typhimurium DT 8

**DOI:** 10.1371/currents.outbreaks.2c05a47d292f376afc5a6fcdd8a7a3b6

**Published:** 2015-02-10

**Authors:** Philip M Ashton, Tansy Peters, Linda Ameh, Ralph McAleer, Stewart Petrie, Satheesh Nair, Ivan Muscat, Elizabeth de Pinna, Tim Dallman

**Affiliations:** Public Health England; Public Health England, London, UK; Public Health England, London, UK; Environmental Health, Public Health Department Jersey, Jersey; Environmental Health, Public Health Department Jersey, Jersey; Public Health England, London, UK; Departments of Microbiology and Public Health, Jersey; Public Health England, London, UK; Public Health England, London, UK

**Keywords:** duck eggs, food-borne infection, outbreaks, phylogeny, Salmonella

## Abstract

Background: Salmonella enterica serovar Typhimurium DT8 is uncommon within the European Union. An increase in this phage type was reported in the summer of 2013 in the States of Jersey. Methods: A total of 21 human cases with this phage type were microbiologically confirmed. Salmonella isolates from mayonnaise made using raw eggs were also confirmed as being Salmonella Typhimurium DT8. The epidemiological investigations strongly supported a link between mayonnaise consumption and illness. Whole genome sequencing (WGS) was used to retrospectively investigate this outbreak with a view to assess the similarity between the suspect food and the human isolates and to characterise a known point source outbreak to assist in development of algorithms for outbreak detection. Results: Sequence data showed that the outbreak associated isolates, including the food isolates, formed a tightly clustered monophyletic group, with a maximum pairwise distance of 3 single nucleotide polymorphisms. Conclusions: WGS data is useful in confirming the causative agent of outbreaks where food and clinical isolates are available. This dataset, comprising a known outbreak, will be useful in the development of automatic algorithms for outbreak detection.

## Introduction

Within the European Union (EU), due to their relative frequency, two particular* Salmonella* ** *enterica *serovars are considered to be of especial public health significance. Together, *Salmonella* Enteritidis (*S*. Enteritidis) and *Salmonella* Typhimurium (*S.* Typhimurium) account for approximately 80% of all the reported human isolates (EFSA, 2009a; 2010a). Although *S.* Enteritidis remains the principal *Salmonella* serovar associated with infections linked to eggs and egg products in the EU, other serovars have also been implicated in egg associated outbreaks, most notable among these is *S*. Typhimurium^1^.

Whole genome sequencing (WGS) has been widely used to investigate outbreaks of a variety of gastrointestinal pathogens, including *Salmonella enterica *serovar Montevideo^5^ , *Salmonella enterica *serovar Typhimurium^6^ and *Shigella sonnei* 7 . At Public Health England (PHE) we are beginning routine WGS of all presumptive *Salmonella enterica* received by the reference laboratory. It is hypothesised that the increased resolution of WGS can be used to detect outbreaks and provide insight into the epidemiology of known outbreaks.

The outbreak described here was defined using existing typing methods (i.e. serotyping and phage typing), however, characterising known outbreaks with WGS will assist in interpretation of routine sequencing data in future outbreaks. Here, we describe the outbreak investigation, as well as the *post hoc *WGS analysis which may be informative for future, prospective use of WGS for outbreak detection.

## Methods

**Microbiology and sample selection**

A total of 21 human outbreak isolates were typed using the White-Kauffmann-Le Minor scheme^8^ and the Anderson *et al* 9 Typhimurium typing phages. There were also five colony picks from the same isolation plate that came from a suspect food isolate (Table 1). For background, 35 sporadic DT 8 isolates from 2012-2014 were also sequenced. DNA was extracted using the QiaSymphony DSP DNA Mini Kit and a Qiagen Automated Extractor.

The antibiotic susceptibility testing was done using breakpoint concentrations. Briefly it is a agar dilution method using Iso-sensitest agar or Muller Hinton agar to determine if the isolate is sensitive or resistant to a set concentration of the antimicrobial. The antimicrobial concentrations used for screening of resistance were: ampicillin 8mg/L, chloramphenicol 8 and 16mg/L, colomycin 2mg/L, sulphonamide 256mg/L, gentamicin 2mg/L, tobramycin 8mg/L, amikicin 8mg/L, streptomycin 16mg/L, tetracycline 8mg/L , trimethoprim 2mg/l , nalidixic acid 16mg/L, ciprofloxacin 0.064 and 0.5 mg/L, ceftazidime 1 and 2 mg/L, cefotaxime 0.5 and 1 mg/L, cefoxitin 8 mg/L, cefpirome 8mg/L , ertapenem 0.064 and 0.5 mg/L, and temocillin 128 mg/L.

**Table 1. Isolate list and associated meta-data (asterix denotes outbreak associated):** 

MolisID	PHE Centre	Receipt date	Sample type	R-type ($ = sensitive to all tested; otherwise mg/L sensitivity)
H121600325	West Midlands	16/04/2012	Human	$
H122720573	Wales	03/07/2012	Human	$
H123920661	Wales	25/09/2012	Human	$
H123020544	Thames Valley	24/07/2012	Human	$
H122020454	Northern Ireland	15/05/2012	Human	$
H124860455	London	29/11/2012	Human	$
H132020501	Lincolnshire, Leicestershire, Nottinghamshire and Derbyshire	14/05/2013	Human	$
H133000654	Jersey*	22/07/2013	Human	$
H133060375	Jersey*	25/07/2013	Mayonnaise	$
H133060376	Jersey*	25/07/2013	Mayonnaise	$
H133060377	Jersey*	25/07/2013	Mayonnaise	$
H133060378	Jersey*	25/07/2013	Mayonnaise	$
H123100316	Greater Manchester	30/07/2012	Human	$
H132460180	Devon, Cornwall and Somerset	13/06/2013	Human	$
H133040470	Anglia and Essex	24/07/2013	Human	$
H130720555	Yorkshire and Humber	12/02/2013	Human	$
H131960204	Thames Valley	09/05/2013	Human	$
H133380323	Thames Valley	16/08/2013	Human	$
H132780266	Sussex, Surrey and Kent	05/07/2013	Human	$
H132960590	Sussex, Surrey and Kent	18/07/2013	Human	$
H131920702	Northern Ireland	07/05/2013	Human	$
H132800431	Northern Ireland	08/07/2013	Human	$
H132980531	Northern Ireland	19/07/2013	Human	$
H133460540	Northern Ireland	22/08/2013	Human	$
H132920685	London	16/07/2013	Human	$
H133260293	Lincolnshire, Leicestershire, Nottinghamshire and Derbyshire	08/08/2013	Human	$
H133400611	Lincolnshire, Leicestershire, Nottinghamshire and Derbyshire	19/08/2013	Human	$
H132940743	Jersey*	17/07/2013	Human	$
H132940744	Jersey*	17/07/2013	Human	$
H132940745	Jersey*	17/07/2013	Human	$
H132940746	Jersey*	17/07/2013	Human	$
H132940747	Jersey*	17/07/2013	Human	$
H132940748	Jersey*	17/07/2013	Human	$
H132940749	Jersey*	17/07/2013	Human	$
H132940750	Jersey*	17/07/2013	Human	$
H132940751	Jersey*	17/07/2013	Human	$
H132940753	Jersey*	17/07/2013	Human	$
H132940754	Jersey*	17/07/2013	Human	$
H132940756	Jersey*	17/07/2013	Human	$
H133000645	Jersey*	22/07/2013	Human	$
H133040472	Jersey*	24/07/2013	Human	$
H133040474	Jersey*	24/07/2013	Human	$
H133300609	Jersey*	12/08/2013	Human	$
H131660213	Devon, Cornwall and Somerset	18/04/2013	Human	$
H132040425	Cumbria and Lancashire	15/05/2013	Human	$
H141620522	Cumbria and Lancashire	15/04/2014	Human	$
H133220558	Cheshire and Merseyside	06/08/2013	Human	$
H130700419	Avon, Gloucestershire and Wiltshire	11/02/2013	Human	$
H133480564	Avon, Gloucestershire and Wiltshire	23/08/2013	Human	$
H133000653	Jersey*	22/07/2013	Human	AMP8, SUL256, STR16, TET8, FOX8
H133340322	Yorkshire and Humber	14/08/2013	Human	COL2
H132300541	Jersey*	03/06/2013	Human	COL2, STR16, TET8
H133060374	Jersey*	25/07/2013	Mayonnaise	NAL16, CIP0.064
H132940752	Jersey*	17/07/2013	Human	STR16
H133040473	Jersey*	24/07/2013	Human	SUL256, STR16

**Sequencing**

In addition to the outbreak associated strains that were sequenced as part of this investigation, an additional 35 S. Typhimurium DT 8 isolates from 2013 (n = 28) and 2012 (n = 7) were also sequenced (Table 1). The DT 8 strains sequenced here were compared with the wider diversity of *S. *Typhimurium sequenced as part of the routine adoption of WGS within PHE. Sequencing was carried out by the PHE Genome Sequencing Unit using Nextera library preparation and the Illumina HiSeq 2500 in fast run mode according to manufacturers’ instructions. FASTQ reads from all sequences in this study can be found at the PHE Pathogens BioProject at NCBI (PRJNA248792).

**Bioinformatics**

Raw FASTQs were processed with Trimmomatic 10 with bases removed from the trailing end that fall below a PHRED score of 30. Sequence typing was performed using a variant of SRST2^17^ . These processed reads were mapped to the *S. *Typhimurium LT2 reference genome (GenBank: AE006468) using BWA mem^11^. SNPs were then called using GATK2^12^ in unified genotyper mode. Core genome positions that had a high quality SNP (>90% consensus, minimum depth 10x, GQ >= 30, MQ >=30) in at least one strain were extracted and FastTree 13 used to derive an approximate maximum likelihood tree for all *Salmonella* Typhimurium and Mega v5^14^ was used to derive the maximum likelihood phylogeny of the DT8 isolates using the Jukes-Cantor substitution model.

## Results

In the summer of 2013, an increase of *S.* Typhimurium was reported in the States of Jersey (a British Crown dependency). In total 21 isolates were sent by the States of Jersey Pathology Laboratory to the Gastrointestinal Bacteria Reference Unit (GBRU) between July 25^th^ and August 27^th^, 2013, exceeding what would be expected from the numbers previously reported in this geographical region.

Food histories from initial cases reported around the 12th July suggested a link with two social events on consecutive days in early July 2013, guest lists for these events were obtained and over 80 people interviewed. The time to onset after attendance at one of these two events was determined (Figure 1). Epidemiological investigations showed that both events were supplied by a business based on a farm which supplied hog roast and accompanying salads using home-made mayonnaise. The original batch of mayonnaise had been completely exhausted in the events, but a subsequent batch was sampled by the Public Health England Wessex Environmental Microbiology Service in the week following the outbreak and this tested positive for* Salmonella*. Microbiological typing at the reference laboratory showed that all isolates, both from the patients and implicated food stuff, were the same definitive phage type (DT8) of *Salmonella *Typhimurium. This provided microbiological evidence to support the epidemiological evidence suggesting the farm as the source of infection.

**Epidemic curve:**
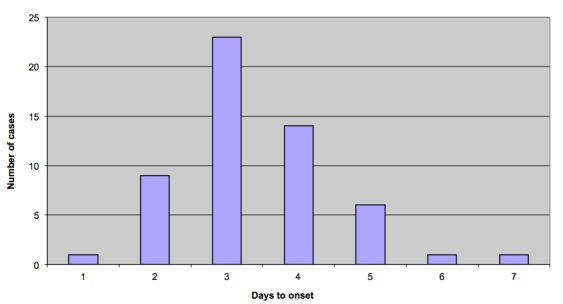
Epidemic curve of *Salmonella *Typhimurium DT 8 outbreak

The farm had a variety of poultry (chickens, ducks, geese, guinea fowl, partridge) as well as pigs. The chickens were inoculated against *Salmonella*, but the ducks and other fowl were not. The mayonnaise was made using whatever eggs were available at the time and normally involved both chicken and duck eggs. Local environmental health officers subsequently addressed issues at the farm and the business is no longer trading. The case was the subject of a successful prosecution in the Royal Court.

A total of 61 isolates were sequenced, 26 linked to the outbreak and 35 background DT 8 isolates to provide context. All outbreak isolates were confirmed as sequence type (ST) 19, a common *S. *Typhimurium ST. Furthermore, these isolates could be put into the context of 412 Salmonella Typhimurium isolates for which WGS had also been performed. The DT 8 isolates formed a monophyletic group within the *S.*Typhimurium isolates sequenced by PHE until June 2014 (n=473) (Figure 2). The DT 8 clade was approximately 2000 SNPs from the LT2 reference genome.

**Salmonella Typhimurium:**
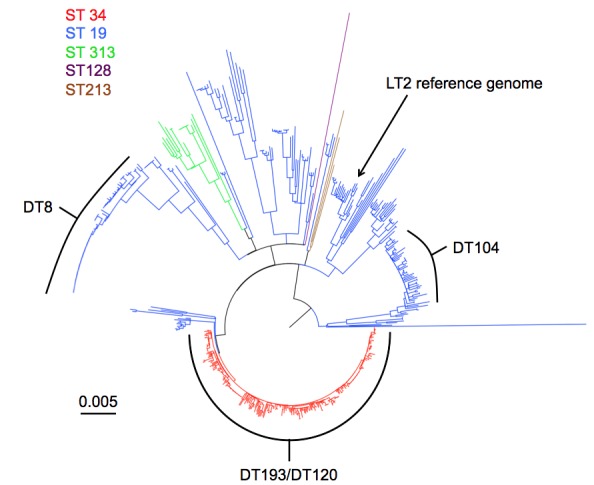
Phylogenetic tree of Salmonella Typhimurium sequenced by PHE until June 2014. Sequence type is denoted by colour. The most common definitive phage types are indicated.

Within the DT 8 clade (Figure 3), there were a total of 342 variable positions between the DT 8 strains, with the most diverse pair of strains having 106 SNPs between them, indicating that while clonal, there is significant diversity within it. All of the outbreak associated isolates, including the five colony picks from the food isolate formed a tightly clustered monophyletic group, with a maximum pairwise distance of three SNPs. Of the five isolates that were from the same food sample, four had no SNPs between them, while one of the isolates was a single SNP away from the others.The closest strain to the known outbreak strains was a DT 8 isolate received by the reference laboratory on 18^th^April 2013 from Plymouth. This isolate was only five SNPs from the closest outbreak strain. The next two closest isolates were eight and 10 SNPs distant from the outbreak. These fell within or close to the temporal range of the known outbreak cases but were geographically separate (London and Anglia & Essex).

**Salmonella Typhimurium DT 8:**
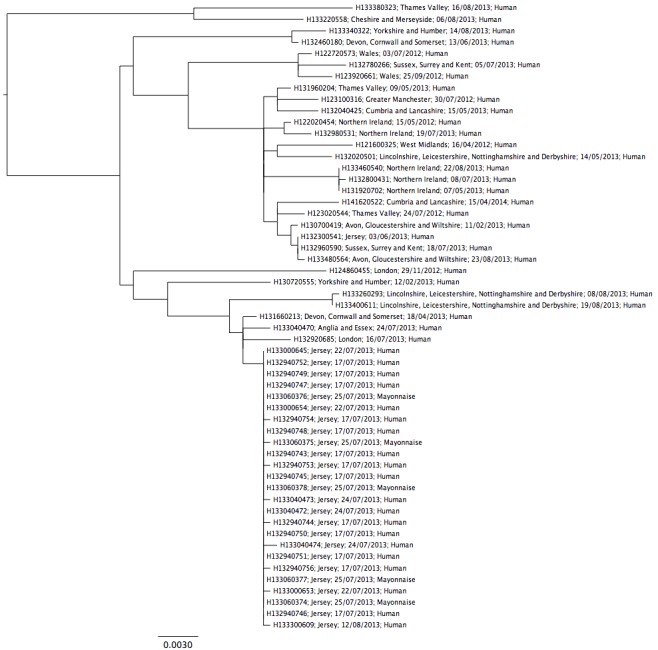
Detailed phylogeny of Salmonella Typhimurium DT 8 sequenced by PHE until June 2014. Annotated with Laboratory reference, region of origin, receipt date, isolation source.

## Discussion

*Salmonella *remains a major cause of food poisoning in the UK and has been associated with both sporadic cases and larger outbreaks. Phage typing is a phenotypic method traditionally used for surveillance and subtyping of salmonellae but is performed in only a few laboratories due to the requirement for standardised phage panels. Subsequently, DNA fingerprinting techniques such as pulsed-field gel electrophoresis (PFGE) or multi-locus variable-number tandem-repeat analysis (MLVA), are often employed reactively in outbreak investigations to supplement phage typing data where further strain discrimination is required. Routine surveillance by WGS methods provides the opportunity for forensic typing to be deployed on all isolates thereby providing the potential to detect linked cases earlier in the outbreak.

This outbreak was caused by DT 8, although this is an uncommon phage type within the EU it has previously been implicated in sporadic cases of salmonellosis during the 1990s associated with consumption of duck eggs in Germany^2^ and was the causative agent for a small salmonellosis outbreak in Scotland in 2009^3^ . It was also the cause for a number of human infections associated with duck eggs that occurred in Ireland between 2009 and 2011^4^ and a UK outbreak associated with duck eggs in 2010^1^. There is no sign that DT 8 is entering poultry flocks in the UK, with only 6 isolations in the past 5 years (AHVLA/APHA Salmonella in Livestock production 2013).

The focus of this study was to identify the number of SNP differences within a known outbreak, where isolates were obtained from the associated food vehicle so to investigate the similarity within and between the suspect food and the clinical cases

For any outbreak investigation, making a linkage between clinical isolates and possible food sources requires distinguishing the suspected pathogen from the circulating background population whatever methodologies are employed. We identified the number of SNP differences within this outbreak of *Salmonella enterica *to be much lower than that identified within previous outbreaks of *Salmonella* characterised by WGS^15^. Previous studies identified more diversity within three of the six outbreaks they investigated than we observed across the entire DT8 phage type. This kind of study to investigate variation in the number of SNPs found in outbreaks emphasises the need for test datasets to be adopted for validation of SNP detection algorithms.

WGS is increasingly being mooted as a molecular epidemiologic tool for trace-back of bacterial pathogens through the food chain. The observation that WGS can determine the clonal nature of an outbreak strain, both from clinical samples and implicated food-stuffs has positive implications for reference microbiology and outbreak investigations.

The fact that there was an isolate within five SNPs of the outbreak that was temporally distinct from the outbreak strains is a reminder that we cannot rely solely on genomic information for determining outbreaks and that basic and enhanced epidemiology will both be invaluable as we enter this exciting new phase of public health microbiology.

As WGS enters routine public health microbiology, there will inevitably be pressure to determine whether or not the diversity within a group of strains indicates a common source of infection. However, this diversity will depend on the diversity present at the source of infection. If we assume that each outbreak is, in the final analysis, caused by a single population, then the diversity within the outbreak will be a sample of the diversity present in that population. Diversity increases with effective population size^16^ . We hypothesise that the larger the underlying population is, the larger the effective population size has the potential to be. Therefore larger facilities would tend to cause outbreaks with greater diversity than smaller facilities. Thus it is not possible to set a single diversity threshold we would expect to see within all *Salmonella* outbreaks because it would depend on the size of the population that caused it. There is a paucity of information studying the relationship at the genomic level between the diversity of *Salmonella *strains seen within different populations. This knowledge gap needs to be filled so we can interpret the diversity observed for *Salmonella *in England and Wales.

In the very near future, a combined approach using epidemiological, clinical, environmental and WGS information will be invaluable in routinely elucidating and detecting outbreaks of *Salmonella* food poisoning.
